# Study on the Quasi-Ductile Fracture Behavior of Glubam: The Role of Fiber Distribution

**DOI:** 10.3390/ma17071611

**Published:** 2024-04-01

**Authors:** Haolei Jiang, Wen Liu

**Affiliations:** Department of Civil Engineering, Beijing Forestry University, Beijing 100083, China; jiang123@bjfu.edu.cn

**Keywords:** fibrous composites, glubam, fiber distribution, quasi-ductile fracture, fracture parameters, staggered structure

## Abstract

Cracking in fibrous composites is inevitable, and the fracture pattern is influenced by its fiber distribution. Bamboo fibrous composites have a distinct fiber distribution, which makes them an excellent material for studng the relationship between fiber distribution and fracture mode. Glued laminated bamboo is a bi-directional bamboo fibrous composite, which is called glubam for short. Its vertical thickness is about 28 mm, and the ratio of the number of longitudinal fiber layers to the number of transverse fiber layers is 4:1. This study conducted three-point bending fracture tests on single-edge notched specimens of glubam to investigate its mode-I fracture characteristics in the transverse vertical direction. The deformation curves show that the specimens still have the load-carrying capacity after reaching the maximum load, and the load shows a trend of step-like decrease, exhibiting a quasi-ductile fracture behavior. Overall, the fracture process can be divided into four stages, including linear, softening, quasi-ductile, and failure stages. In this study, based on certain assumptions, the prefabricated notch length *a*_0_ was adjusted according to the position of the transverse fibers. Subsequently, the non-linear elastic fracture mechanics method was employed to calculate the fracture parameters of glubam during the softening and quasi-ductile stages, including the fracture toughness K_IC_* and fiber tensile strength *f_t_*. The deviation of the fracture parameters between the two stages is within 10%, indicating that the correction of the *a*_0_ is correct. This indirectly proves that the staggered structure formed by longitudinal and transverse fibers is responsible for the quasi-toughness fracture of glubam. Finally, this study summarized and analyzed the quasi-ductile fracture behavior and found that materials or structures exhibiting quasi-ductile fracture behavior often possess a staggered structure. This staggered structure makes the crack in the form of semi-stable propagation, while the load decreases in a step-like manner.

## 1. Introduction

Bamboo is a natural fibrous composite that has excellent mechanical properties [1,2,3,4,5,6,7,8] and is commonly referred to as “plant bar” [9,10]. However, bamboo has some defects because of its hollow cross-section, fixed nodes, etc., which lead to limitations in its engineering applications [11]. To solve this problem, engineered bamboo made from natural bamboo was born. Glue bamboo is an engineered bamboo product which not only has good biodegradability and low processing energy consumption but also retains the excellent properties of natural bamboo, with low density, high strength, and high toughness. In addition, it also has good plasticity, and its shape can be freely adjusted according to engineering requirements. Li et al. [12] reported the strength values of glued bamboo under 15 different loading conditions, comparing them with C40 wood from the EU, 2850FB-2.3E wood from North America, and SG15 wood from New Zealand, finding that the strength of glued bamboo is significantly higher than these woods. Liu Sharma et al. [13] analyzed the material characteristics of glued bamboo, Sitka spruce, and laminated veneer lumber (LVL). They found that glued bamboo exhibits higher compressive and tensile strength, while its bending performance is similar to that of Sitka spruce and LVL. It can be seen that glued bamboo has broad application prospects in the engineering field, and it can even substitute traditional wood.

**Figure 1:**
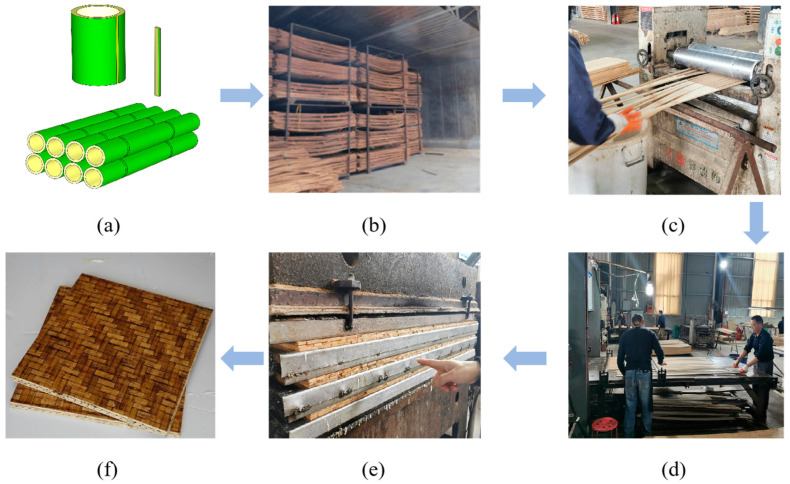
Production process of glubam: (**a**) splitting into strips; (**b**) air-dried at room temperature; (**c**) bamboo strip gluing; (**d**) prepared into veneer; (**e**) hot pressing into boards; (**f**) glubam boards.

According to the different fiber distribution, glue bamboo can be divided into unidirectional and bi-directional glue bamboo. Glued laminated bamboo (Glubam) is a bi-directional bamboo fibrous composite with longitudinal (L-direction) fibers and transverse (T-direction) fibers and four times as many layers of longitudinal fibers as transverse fibers [14]. Figure 1 shows the production process of glubam, including bamboo selection and slicing, air-dried at room temperature, bamboo strip gluing, veneer production, hot-pressing, and post-processing. Up till now, the mechanical properties of glubam have been widely researched. For example, Xiao et al. [15] were the first to investigate the mechanical properties of glubam broad, and they obtained basic mechanical properties such as compressive strength, elastic modulus, and bending strength. Xiao et al. [16] also reported the in-plane and out-of-plane shear strength of glubam and gave the corresponding failure mechanism. Yang [17] improved the Hankinson formula on the basis of a series of assumptions. The improved Hankinson formula can better predict the off-axis tensile strength of glubam. Table 1 gives the mechanical strength of glubam parallel to fiber direction obtained from the previous literature [12,13,14,15,16,17,18,19,20,21,22] and also includes the mechanical properties of laminated bamboo lumber (LBL), Douglas fir, and wood–plastic composite (WPC). The comparison with other bamboo and wood showed that the mechanical properties of glubam are reasonable and with better performance than average. At present, glubam has been applied in various structural systems in engineering, including but not limited to prefabricated panel houses [23], frame houses, trusses [24,25], bridges [26,27], and other constructions.

Bamboo fibers have better mechanical properties only in the length direction [28,29,30,31,32,33,34,35], so there are significant differences in the fracture characteristics of bamboo fibrous composites along and perpendicular to the fiber direction. In addition, this difference is influenced by the distribution of fibers, showing a more complex mode of variation. The fracture of bamboo along the fiber direction is generally of the brittle fracture mode [36,37,38]. When bamboo cracks, the crack propagates at the interface between the fiber and the matrix, forming an inter-layer fracture. The commonly used test methods are the double cantilever beam method and the compact tension method [39].

Bamboo fibrous composites can be divided into unidirectional and bi-directional fibrous composites according to the distribution of fibers. Their fracture characteristics in the direction perpendicular to the fiber are very complicated, which has attracted the attention of many scholars. Natural bamboo is a unidirectional bamboo fibrous composite with uneven fiber distribution in the cross-section. The highest content of fibers is located on the outer layer of the bamboo, followed by the middle layer, and with the least amount on the inner layer [40,41]. Usually, cracks first develop inside the bamboo and then extend toward the middle part. When encountering an area with dense fibers, cracks will deflect and propagate along the fiber direction until the load increases to the fibers break. The crack propagates in a tortuous manner, which makes the bamboo have high fracture toughness, and its fracture is a non-catastrophic fracture [42]. Bamboo scrimber refers to unidirectional fibrous composites with uniformly distributed fibers in the cross-section. Unlike natural bamboo, its fracture mode belongs is that of quasi-brittle fracture [43,44]. Glubam is bi-directional bamboo fibrous composites. Liu et al. [45] reported its fracture behavior using the Arcan test method. The test results show that the load drops sharply after reaching the maximum load, followed by a long and slow decline stage. This indicates that the fracture of glubam is non-catastrophic. However, they only found that transverse fibers could improve glubam fracture behavior but did not study it in depth.

To further investigate the effect of fiber distribution on the fracture behavior of fibrous composites, this study analyzed the fracture process of glubam based on the 3-p-b fracture test and calculated its fiber tensile strength *f_t_* and fracture toughness K_IC_*. The role of transverse fiber in the fracture of glubam was investigated through fracture parameters.

## 2. Materials and Methods

### 2.1. Test Material

**Figure 2:**
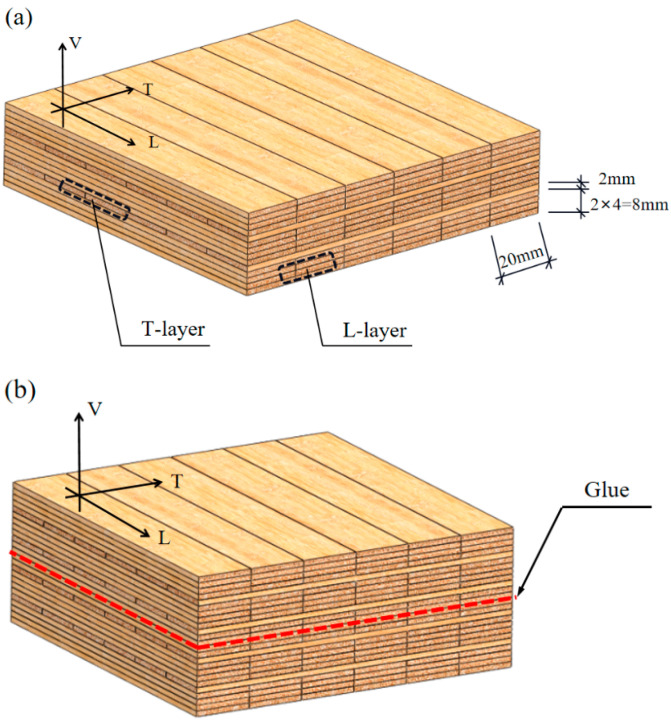
Bidirectional fiber glubam boards for fracture test: (**a**) two-T-layer board; (**b**) four-T-layer board.

In this study, glubam was made from 4-year Moso bamboo grown in Hunan Province, China. The commercial glubam board is made of 20 mm-wide and 2 mm-thick bamboo strips, with an overall size of 1220 mm × 2440 mm and an overall thickness of approximately 28 mm. In order to investigate, in depth, the role of transverse fibers in glubam fracture, this study vertically glued two commercial glubam boards together to form a composite board with four layers of transverse fibers, which is 48 mm thick, as shown in Figure 2. For convenience, the transverse fiber layer can be named the T-layer, and the longitudinal fiber layer can be named the L-layer. According to the number of T-layers contained in the board, the test boards can be named the two-T-layer board and four-T-layer board, respectively. The moisture content of the glubam is 6.5% and its density is 840.5 kg/m^3^.

### 2.2. Test Method

**Figure 3:**
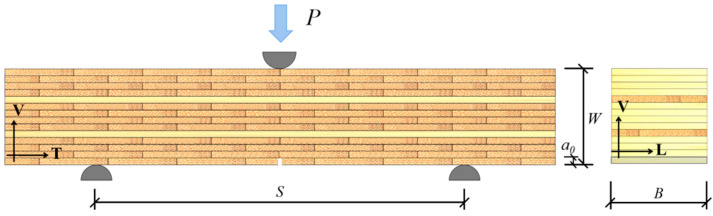
Geometry of SEN specimens taken from 2-T board.

This study uses the 3-p-b fracture test method to study the TV direction fracture mechanism of glubam. The geometry of the single-edge-notched (SEN) specimen taken from the 2-T plate is shown in Figure 3, where *S* is the span, *B* and *W* are the width and thickness of the specimen, respectively, and *a*_0_ is the prefabricated notch length. Six or seven specimens were prepared for each group. The SEN specimens were grouped according to the board type and the number of fiber layers that the prefabricated notch penetrates, and the grouping results are shown in Table 2. The 3-p-b fracture test was carried out by a WDW-100E testing machine. To obtain a stable load–deflection curve during the test, the displacement loading rate was 2 mm/minute to ensure that the specimens fully developed their mechanical properties before fracture. The prefabricated notch was made by an electric curve saw with a width of 1 mm.

In Group No., G_2_ and G_4_ refer to a two-T-layer board and a four-T-layer board, respectively; L refers to the L-layer; T refers to the T-layer; and the number is the number of fiber layers; for example, G_4_-L4T1 refers to a four-T-layer board specimen with a prefabricated notch that penetrates four L-layers and one T-layer.

## 3. Test Results

### 3.1. Fracture Crack Modes

**Figure 4:**
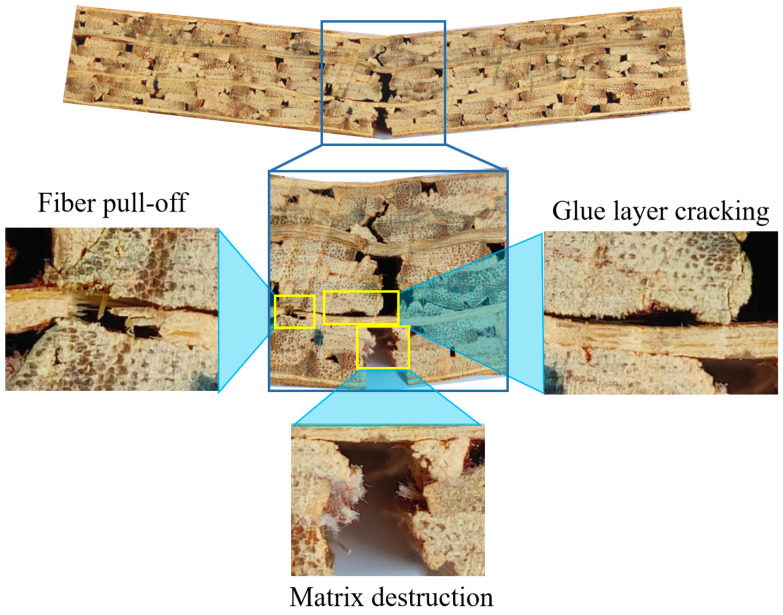
Failure modes of specimens of G_2_-L1 group in bending process.

Figure 4 shows the failure mode of specimens from the G_2_-L1 group during the bending process. The crack starts at the tip of the prefabricated notch and the overall propagation direction is in the loading direction. There are three damage modes in the specimen, namely, matrix destruction, fiber pull-off, and glue layer cracking. These damage modes consume the energy of crack propagation and effectively improve the toughness of glubam.

**Figure 5:**
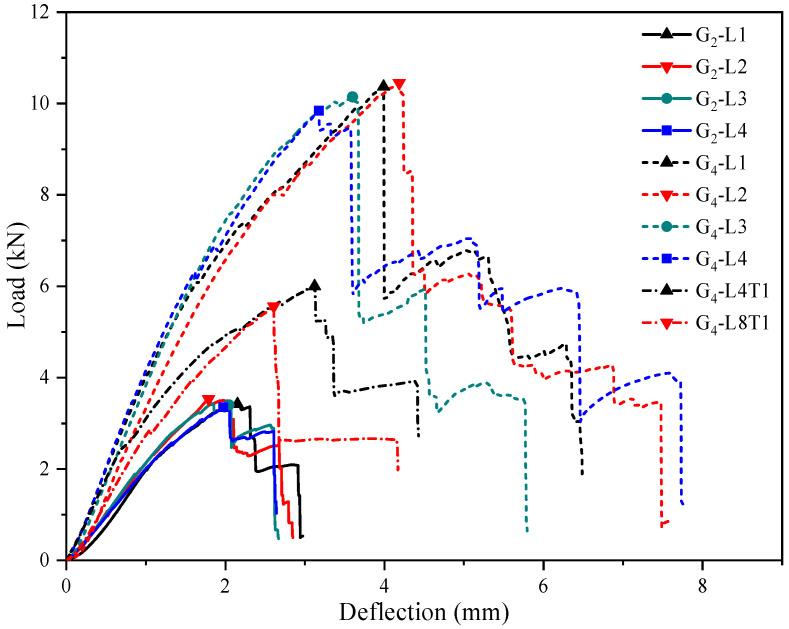
The representative load–deflection curves for each group.

Figure 5 shows the representative load–deflection curves for each group. All the deformation curves are extremely similar. First, they all have a linear stage and softening stage. Second, they all have a steep load drop and rise after reaching the maximum load. Finally, they all end the change in the failure stage. According to the maximum load, the deformation curves can be divided into three groups: 2-T group, 3-T group, and 4-T group. The specimens in the 2-T group are made of two-T-layer boards and contain two layers of transverse fibers. The specimens in the 3-T group are made of four-T-layer boards but only contain three layers of transverse fibers. The specimens in the 4-T group are made of four-T-layer boards and contain four layers of transverse fibers. 

### 3.2. Quasi-Ductile Fracture Process

**Figure 6:**
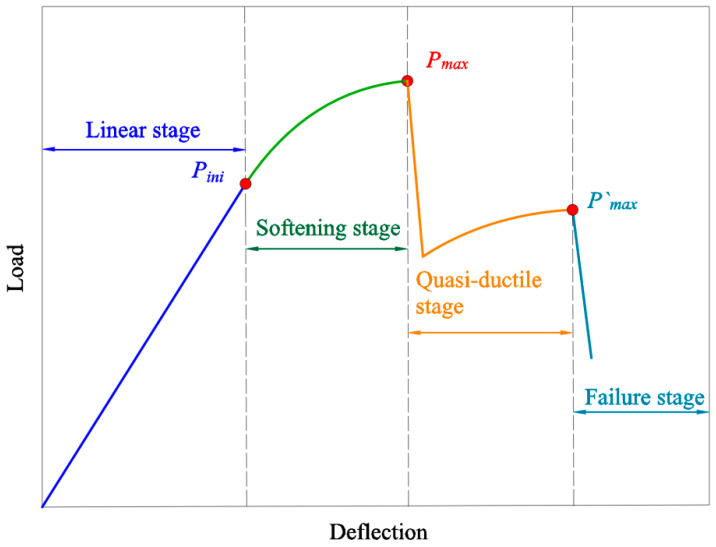
Deformation curve of two-T-layer board.

**Figure 7:**
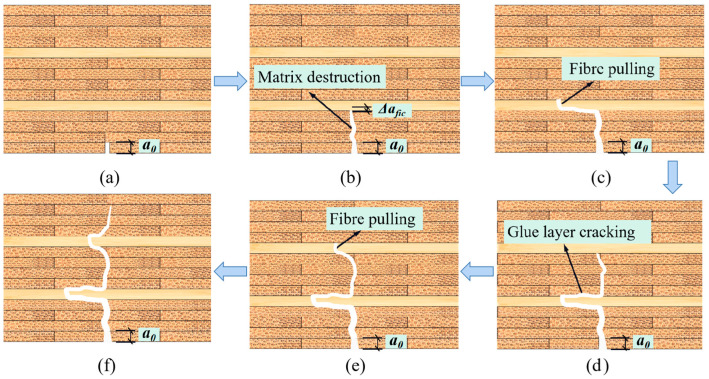
Crack development process: (**a**) linear stage; (**b**) softening stage; (**c**,**d**) quasi-ductile stage; (**e**,**f**) failure stage.

Figure 6 shows the idealized deformation curve of specimens in the 2-T group. Figure 7 shows an idealized schematic of the crack extension form of the specimen in Figure 4. As shown in Figure 6, the fracture process of glubam can be divided into four stages, including linear stage, softening stage, quasi-ductile stage, and failure stage. The division points of the four stages correspond to the initial cracking load *P_in_*_i_ of the linear stage, the maximum load *P_max_* of the softening stage, and the complete fracture load *P′_max_* of the quasi-ductile stage, respectively.

First, in the linear stage, glubam has a certain elastic strain; the increase in deformation does not cause the material to generate micro-cracks, as shown in Figure 7a. At this stage, the applied load is relatively small, and the stress has not reached the material’s elastic limit stress. Therefore, the load–deflection curve is a sloping straight line until the load increases to the material’s initial cracking load *P_in_*_i_.

Second, after the applied load exceeds *P_in_*_i_, the crack begins to develop along the most dangerous path, as shown in Figure 7b. Due to the appearance of cracks, the stiffness of the specimen decreases, resulting in the non-linear relation of the load–deflection curve. At this stage, the crack propagates to the T-layer and produces micro-cracks in the T-layer. As the micro-cracks continuously enlarge, they eventually form through micro-cracks, which are equivalent to the fictitious cracks. When the fictitious crack reaches the critical length Δ*a_fic_*, the applied load arrives at *P_max_*, and at the fibers bottom of the T-layer, macroscopic fracture occurs. This non-linear stage is called the softening stage.

Then, when the first T-layer cracks, the load drops sharply and the macro-crack propagates towards the loading point along the fictitious crack path until the first T-layer is completely fractured, as shown in Figure 7c. However, even if partially fractured, the specimen is able to continue to withstand the load. As shown in Figure 7d, when the load is increased again, the cracks continue to propagate until they propagate to the bottom of the second T-layer. When the crack develops to the bottom of the second layer of transverse fibers, the virtual crack grows in the second layer of transverse fibers until the fiber bundle reaches the tensile stress limit and the specimen reaches the second maximum load *P′_max_*. This stage, in which the crack is in semi-stable propagation while the load step-like decreases, can be called the quasi-ductile stage. This behavior of a sharp drop and rise in the load is repeated in the 4-T group with more T-layers.

Finally, in the failure stage, the cracks penetrate all T-layers, the load drops sharply for the last time, and the specimen loses its load-bearing capacity completely, as shown in Figure 7e,f.

During the fracture of glubam, the load showed a step-like decrease and the cracks showed a semi-stable expansion pattern. This means that the TV direction fracture of glubam represents a non-catastrophic fracture mode and a quasi-ductile fracture behavior.

### 3.3. Comparison between Different Four Fracture Modes

**Figure 8:**
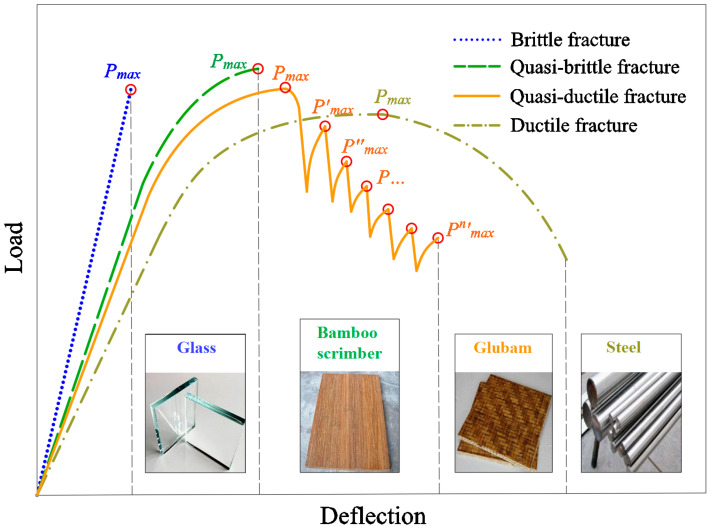
Deformation curves of four fracture modes.

Compared to quasi-ductile fracture, brittle, quasi-brittle, and ductile fracture modes are more common in daily life. Figure 8 shows the deformation curves of four fracture modes. Brittle fracture only has a linear stage, and there is no warning before the fracture occurs. This fracture mode is common in brittle materials such as glass [46,47] and cast iron [48]. Quasi-brittle fracture exists with a softening stage and there is deformation softening before the occurrence of fracture. This fracture mode is common in quasi-brittle materials such as bamboo scrimber [43,44], concrete [49,50], and rocks [51]. Ductile fracture has obvious macroscopic plastic deformation, which is the safest fracture mode. This fracture mode is common in ductile materials such as steel [52,53] and high-density polyethylene [54]. It can be observed that there are significant differences between the quasi-ductile fracture and the other three fracture modes. It can also be found that the deformation curves of quasi-ductile fracture and quasi-brittle fracture before reaching the *P_max_* show some similarity. 

## 4. Fracture Parameters at *P_max_* in Softening Stage

### 4.1. Fracture Parameters Calculation

Interestingly, fibrous composites can show different fracture modes, such as quasi-brittle fracture and quasi-ductile fracture, which are directly related to the distribution of fibers in the material. The fracture behavior of unidirectional fiber composite bamboo scrimber belongs to the quasi-brittle fracture mode, and previous work [43,44,55] has successfully predicted its fracture parameters using a non-linear elastic fracture mechanics non-LEFM method (Equation (1)). Based on the similarity between quasi-ductile fracture and quasi-brittle fracture, this study also uses this model to calculate the fracture parameters of glubam. Particle size is an important parameter in calculating fracture parameters using this model. Obviously, the structure of glubam does not contain granular aggregates. Referring to previous studies [43,44,55], this study analogizes the average fiber diameter *G* of the T-layer to the particle size and takes *G* = 0.4 mm.

It Is noteworthy that when the T-layer of the glubam fractures, the load decreases significantly, among which the drop at *P_max_* in the softening stage and the first drop (*P′_max_*) in the quasi-ductile stage are the most obvious. Therefore, this study focuses on analyzing the fracture parameters under these two conditions.

(1) $$f_{t}=\frac{P_{\max}}{A_{e}\left(W,a_{0},G\right)}=\frac{1.5P_{\max}\left(\frac{S}{B}\right)\sqrt{1+\frac{a_{e}}{a_{ch}^{*}}}}{\left(W-a_{0}\right)\left(W-a_{0}+2\Delta a_{fic}\right)}=\frac{1.5P_{\max}\left(\frac{S}{B}\right)\sqrt{1+\frac{a_{e}}{0.5G}}}{\left(W-a_{0}\right)\left(W-a_{0}+3G\right)}$$

(2) $$K_{IC}*=2f_{t}\times \sqrt{a_{ch}^{*}}$$

In Equation (1), *f_t_* is the fiber tensile strength, and *A_e_* is the equivalent area function, which is only related to *W*, *B*, S, *a*_0_, and *G*. Obviously, the relationship between *P_max_* and *A_e_* is linear, and this linear relationship has *f_t_* as its slope. Therefore, *P_max_* of the material can be estimated by the fiber tensile strength of the sample. *a_e_* is the equivalent crack length. Using the concept of equivalent crack, *a*_0_ with different sizes can be transformed. a^*^_ch_ is the characteristic crack length, which is related to the *G* and determines the gradual failure transition between the fiber tensile strength *f_t_* criterion and the fracture toughness K_IC_* criterion, namely, Equation (2). Since the fracture toughness in this paper is not obtained based on a linear stage, it is not a *K_IC_* in the strict sense of the term. In this paper, the fracture toughness obtained based on non-linear fracture mechanics is labeled as K_IC_*. Referring to the previous research [43,44,55], take Δ*a_fic_* = 1.5 *G* and a^*^_ch_ = 0.5 *G*.

**Figure 9:**
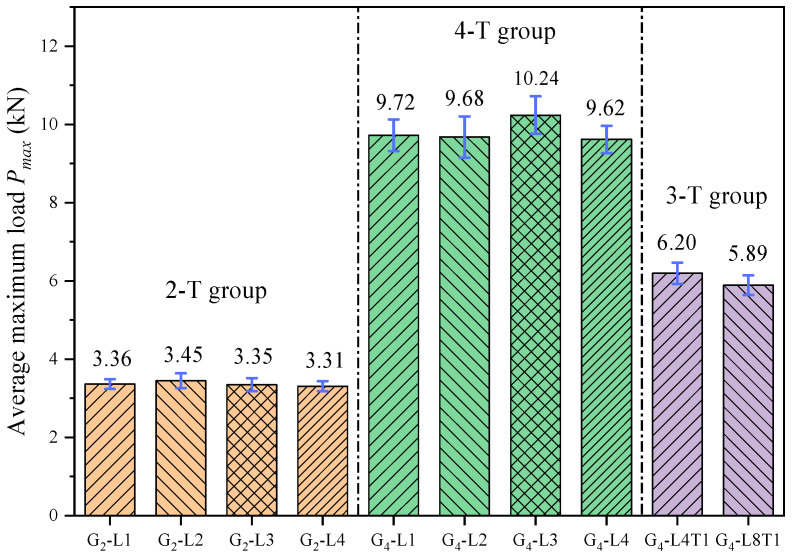
Average maximum load of each group.

As shown in Figure 9, for the 2-T and 4-T groups, the maximum load of the specimens was almost unchanged when the prefabricated notch length was varied from 2 to 8 mm (thickness of the 1–4 fiber layers). In contrast, for the 3-T group (G_4_-L4T1 and G_4_-L8T1), the maximum load of the specimen produces a huge decrease compared to the 4-T group due to the prefabricated notch running through the first transverse fiber layer. Therefore, it can be known that the change in crack length has almost no effect on the maximum load of the specimen when the first T-layer is not damaged. However, in general, *P_max_* should decrease as *a*_0_ increases. Therefore, in order to obtain accurate fracture parameters, an assumption is made in this study that the change in crack length in the L layer has no effect on the maximum bearing capacity of glubam when the cracks do not penetrate the T layer. Therefore, the *a*_0_ entries in Table 2 are altered in this study. For the 2-T group and 4-T group, take *a*_0_ = 4-layer fiber layer thickness; that is, the top of the prefabricated notch is at the bottom of the first T-layer. For the 3-T group, take *a*_0_ = 9-layer fiber layer thickness; that is, the top of the prefabricated notch is at the bottom of the second T-layer. For example, for the G_2_-L1 in 2-T group, although the prefabricated notch length made in the test is 2 mm, only when the crack reaches the transverse fiber layer (at this time, the crack length is the thickness of four fiber layers, which is about 8 mm), does it have an effect on the maximum load. Therefore, in the 2-T group, *a*_0_ is directly taken as the thickness of 4 fiber layers, and at this time, the top of the crack is at the bottom of the first transverse fiber layer.

**Figure 10:**
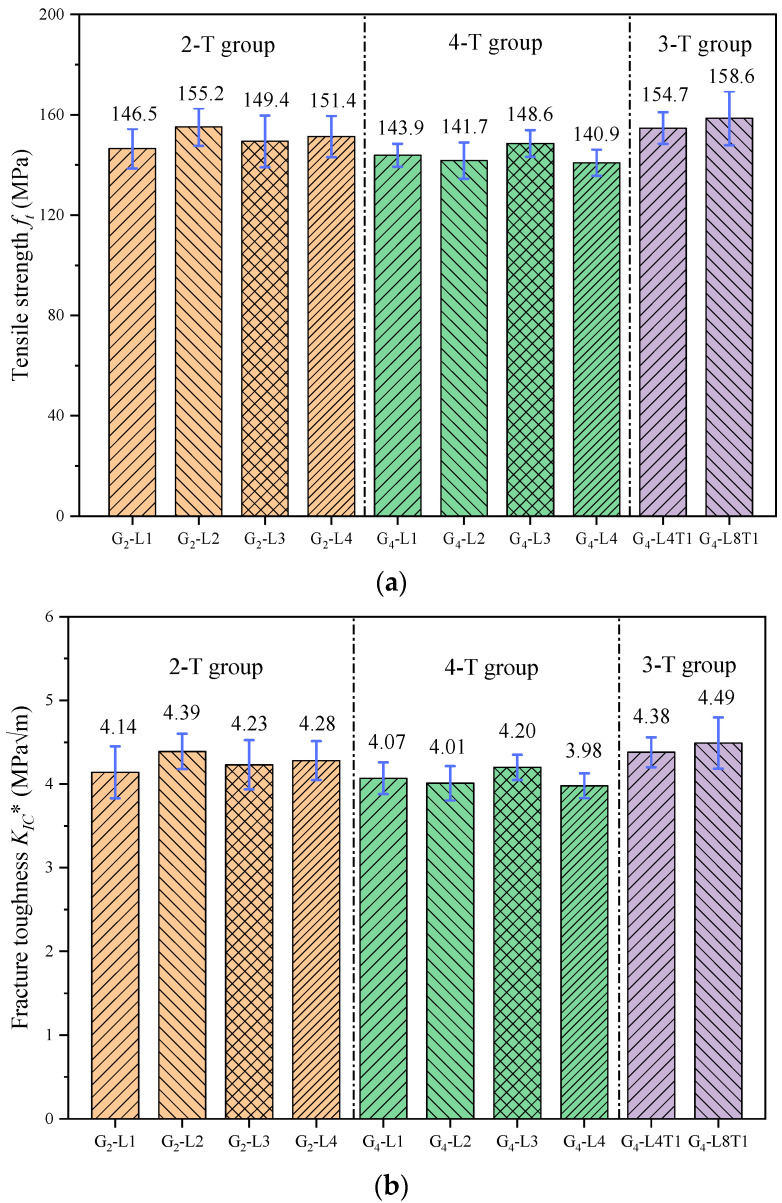
Fracture parameters after modification of *a*_0_: (**a**) tensile strength *f_t_*; (**b**) fracture toughness K_IC_*.

According to Equations (1) and (2), after modifying *a*_0_, the fracture parameters of all the specimens can be obtained. The mean values of fiber tensile strength and mean values of fracture toughness for each group of specimens are given in Figure 10. The fiber tensile strength obtained by previous studies is between 120 and 180 MPa [56], and the results of the experimental calculations are within this range, indicating that the assumptions made in this study are correct.

### 4.2. Statistical Analysis of Fracture Parameters

**Figure 11:**
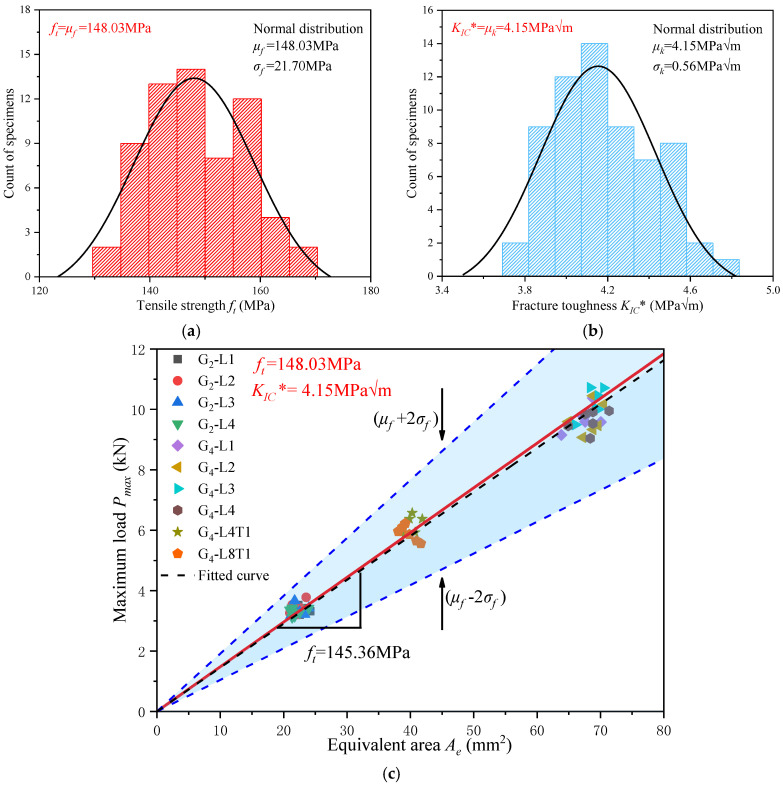
Analysis of fracture parameters at *P_max_* in the softening stage based on normal distribution: (**a**) tensile strength *f_t_*; (**b**) fracture toughness K_IC_*; (**c**) *P_max_*-*A_e_* linear prediction with *f_t_* as slope.

The uncertainty in the testing process and the non-uniform defects in the bamboo result in highly scattered test results. Previous works [43,44,55] have demonstrated the efficacy of normal distribution analysis in fitting fracture parameters of bamboo fiber composites. This study also used the normal distribution analysis method to analyze the fiber tensile strength *f_t_* and the fracture toughness K_IC_* of glubam when the applied load arrives at *P_max_*; the results are shown in Figure 11a,b. It is seen that the fracture parameters predicted by this normal method are *f_t_* = *μ_f_* = 148.03 MPa and K_IC_* = *μ_k_* = 4.15 MPa$\sqrt{m}$. 

The tensile strength *f_t_* of the material is constant. Based on Equation (1), it can be seen that *P_max_* and *A_e_* are a straight line passing through the origin with a slope of *f_t_*. This allows *A_e_* to be obtained while knowing only the size of the specimen, thus predicting the maximum load-carrying capacity of the material. Figure 11c shows the prediction of the fiber tensile strength of glubam with different sizes and different notches by Equation (1). In the three linear *P_max_*-*A_e_* curves, the red line is the mean curve, the slope is *μ_f_* = *f_t_*; and the blue line is the upper and lower boundary with 96 % reliability, the slope is *μ* ± 2*σ_f_*. Clearly, all results are covered by the 96% confidence range, so *f_t_* and K_IC_* can be reliably determined. In addition, a linear fit of the test data yielded the black dash-dotted line in Figure 11c with the fiber tensile strength fit of 145.36 MPa, which has an error of only 1.8% compared to the fiber tensile strength predicted by the normal distribution. This indicates that at the softening stage *P_max_*, the normal distribution analysis method can accurately predict the glubam fracture parameters and cover the experimental data points with sufficient reliability.

## 5. Fracture Parameters at *P′_max_* in the Quasi-Ductile Stage 

### 5.1. Fracture Parameters in the Quasi-Ductile Stage

**Figure 12:**
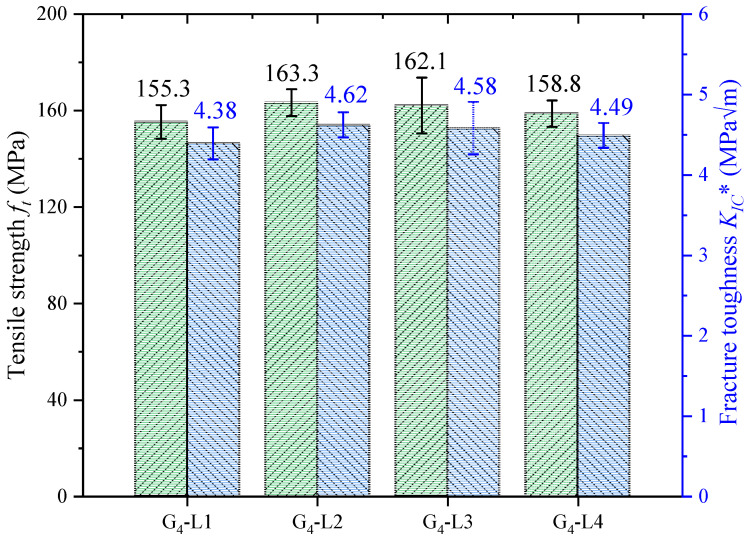
The tensile strength *f_t_* and fracture toughness K_IC_* for 4-T group at *P′_max_*.

It is necessary to analyze the fracture parameters in the quasi-ductile stage. In the quasi-ductile stage, the load shows a step-like decrease and the crack shows semi-stable propagation, and this behavior plays an important role in the safety of glubam in practical applications. In this study, the fracture parameters of the 4-T group under the first maximum load (*P′_max_*) in the quasi-ductile stage are analyzed using Equation (1). When the applied load arrives at *P′_max_*, the crack develops to the bottom of the second T-layer. Thus, *a*_0_ corresponding to is set to nine-layer fiber layer thickness. The fiber tensile strength *f_t_* and the fracture toughness K_IC_* at *P′_max_* of the 4-T group are shown in Figure 12. The calculation results of fiber tensile strength are in the normal range, which shows that the assumptions made in this study are also reliable in the quasi-ductile stage.

### 5.2. Statistical Analysis of Fracture Parameters in the Quasi-Ductile Stage

**Figure 13:**
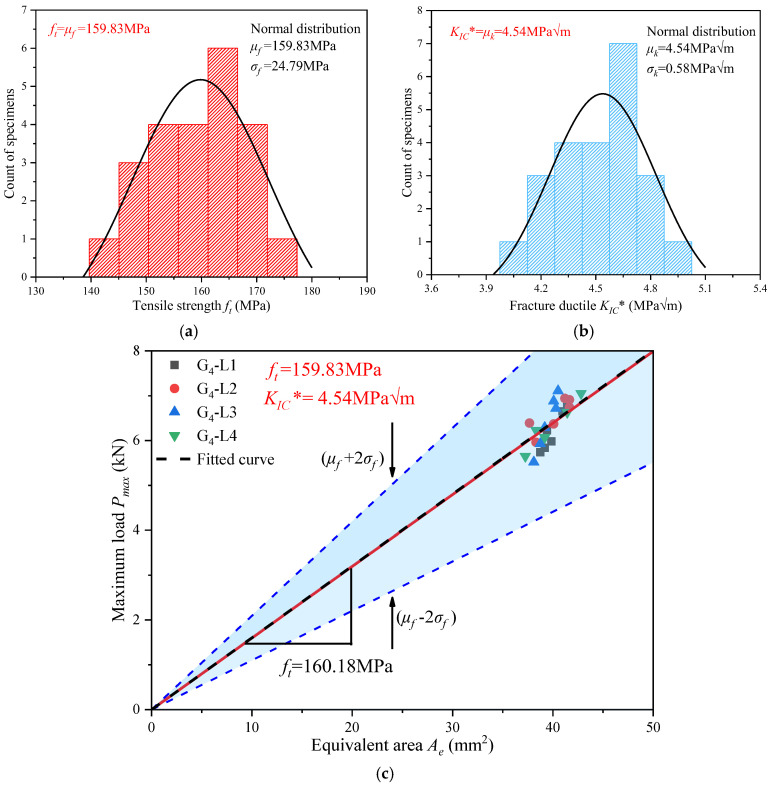
Analysis of fracture parameters in quasi-ductile stage based on normal distribution: (**a**) tensile strength *f_t_*; (**b**) fracture toughness K_IC_*; (**c**) *P_max_*-*A_e_* linear prediction with *f_t_* as slope.

The normal distribution analysis method is also used to fit the fracture parameters of the quasi-ductile stage. As shown in Figure 13a,b, the fiber tensile strength is *f_t_* = *μ_f_* = 159.83 MPa and the fracture toughness is K_IC_* = *μ_k_* = 4.54 MPa$\sqrt{m}$. As shown in Figure 13c, three linear *P_max_*-*A_e_* curves are constructed, and the upper and lower bounds form a confidence range with 96% reliability, which covers all of the experimental scattered points. In addition, a linear fit of the test data yields the black dash-dotted line in the figure with the fiber tensile strength fit of 160.18 MPa, which is almost the same as the fiber tensile strength predicted by the normal distribution. 

It can be seen that although there is some deviation in each group of data, their fiber tensile strength mean values are generally at 140~180 MPa, and the fracture parameters obtained in the quasi-ductile stage differ no more than 10% from those obtained in the softening stage. This indicates that at the quasi-ductile stage *P′_max_*, the normal distribution analysis method can also accurately predict the glubam fracture parameters and cover the experimental data points with sufficient reliability.

### 5.3. Relation of Two-Stage Fracture Parameters

Analyzing the fracture parameters in Figure 11 and Figure 13, it can be seen that the deviation of fiber tensile strength *f_t_* derived from *P_max_* and *P′_max_* is 7.8% and the deviation of fracture toughness K_IC_* is 9.4%. Considering the experimental error and the non-uniform defects of the glubam, this error is acceptable. This shows that as long as the true crack length corresponding to the extreme load is determined, the correct fracture parameters can be obtained using the method used in this paper.

In this study, the L-layer of glubam mainly plays the role of filling, and the T-layer is responsible for load-bearing, both of which form a staggered structure so that the load decreases in a step-like manner, and the maximum load of each step is related to the transverse fiber. In determining the fracture parameters at *P_max_*, the prefabricated notch is taken to the bottom of the first T-layer, and in determining the fracture parameters at *P′_max_* in the quasi-ductile stage, the prefabricated notch is taken to the bottom of the second T-layer, which indicates that the determination of the fracture parameters is related to the fiber distribution of the material. From the selection of *a*_0_ and the variation of the fracture parameters, it can be seen that the quasi-ductile fracture of glubam originates from the staggered structure formed by the longitudinal fiber layers and transverse fiber layers.

## 6. Discussion on Quasi-Ductile Fracture Behavior

In recent years, some researchers have noticed the existence of quasi-ductile fracture behavior in some materials or structures. For example, Zhang et al. [57,58] and Bai et al. [59] found quasi-ductile fracture behavior in different kinds of ceramic materials, respectively, and we also found quasi-ductile fracture behavior in the fracture of glubam [45]. However, these studies only point out that these materials have quasi-ductile fracture behavior; they do not investigate the inherent reasons. Therefore, this study synthesizes previous research results, comprehensively analyzes quasi-ductile fracture behaviors in materials and structures, and elaborates on specific characteristics of quasi-ductile fracture in detail. The following is a detailed discussion of the main characteristics of quasi-ductile fracture. 

### 6.1. Quasi-Ductile Fracture Behavior in Bamboo Fibrous Composites

**Figure 14:**
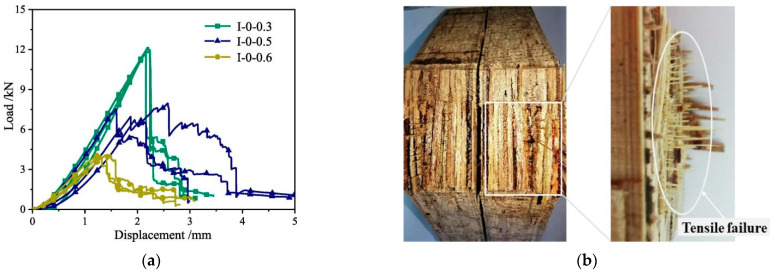
(**a**) Load–deflection curve. (**b**) Fracture surface [45].

The quasi-ductile fracture behavior of glubam is also obvious when it is loaded in TL direction [45]. As shown in Figure 14a, after the steep drop in load, the remaining transverse fibers consume energy through pull-off and pull-out mechanisms, thus enabling the specimen to retain a certain degree of load-bearing capacity. When the load is increased to the ultimate bearing capacity of the transverse fibers, the transverse fibers fracture again, leading to another sudden drop in load. This cyclic process repeats itself, resulting in multiple step-like decreases in the load, until the specimen is eventually destroyed, as shown in Figure 14b. Combined with the performance of the transverse fiber in the test, it can be concluded that the transverse fiber causes the TL fracture of glubam to change from matrix-dominated brittle fracture to fiber-dominated quasi-ductile fracture.

### 6.2. Quasi-Ductile Fracture Behavior in Ceramic Materials

**Figure 15:**
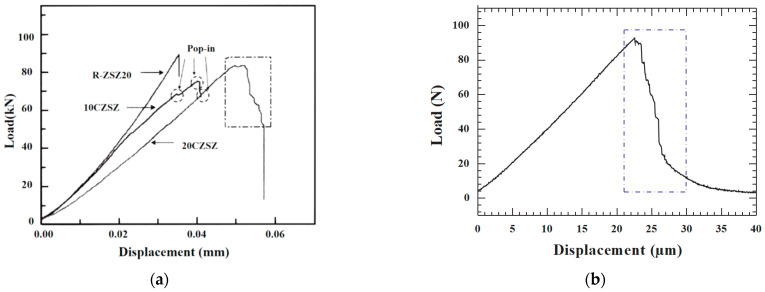
(**a**) Load–deflection curves of 20CZSZ [58]. (**b**) Load–deflection curves of Ti_2_AlC [59].

The fiber-reinforced composites ceramic 20CZSZ (ZrB_2_-SiC-ZrSi_2_ ceramic with 20 vol % short-cut carbon fibers) shows quasi-ductile fracture behavior. Zhang [58], through conducting 3-p-b tests on 20CZSZ and CZSZ samples without short-chopped carbon fibers added, found that 20CZSZ with short-chopped carbon fibers added exhibited quasi-ductile fracture behavior. As shown in Figure 15a, after loading to the maximum load, the load does not drop sharply, but there is a dense oscillation followed by multiple step-like drops until the material fails. This indicates that after the material cracks by reaching the load-carrying limit, the cracks do not rapidly destabilize and expand but behave as a semi-stable expansion form. Through observing the specimen, it can be seen that the cracks deflect along the interface, and a large number of carbon fibers are deboned or pulled out at the cracks. This result demonstrates that the incorporation of carbon fibers changes the fracture behavior of 20CZSZ from brittle to quasi-ductile. Among the ceramic materials, the large-scale Ti_2_AlC [59] shows more obvious quasi-ductile fracture characteristics, as shown in Figure 15b. It particles are board-like, and these board-like particles are distributed in lapped layers, forming a staggered structure. This staggered structure not only reduces the load in a step-like manner, but also greatly blunts the crack tip and has a strong toughening effect.

### 6.3. Quasi-Ductile Fracture Behavior in Masonry Structures

**Figure 16:**
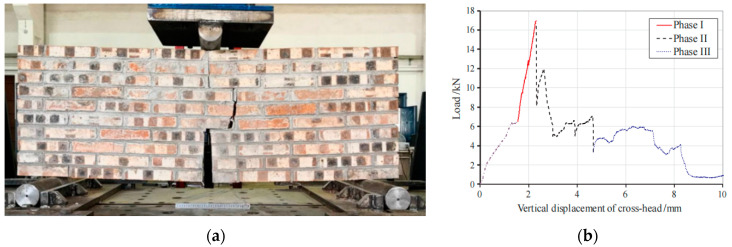
(**a**) Fracture surface of brick wall. (**b**) Load–deflection curve of brick wall [60].

It is interesting that quasi-ductile fracture also exists in masonry structures. As shown in Figure 16a, large brick walls [60] have a rich staggered structure due to the special lap of brick and mortar. This staggered structure enables the brick wall to consume energy through mechanisms such as crack deflection when it reaches its maximum bearing capacity, rather than immediately failing, thereby allowing the wall to maintain a certain degree of load-bearing capacity (Figure 16b). It is obvious that the brick wall fracture represents a quasi-ductile fracture behavior.

From the above description, it can be found that quasi-ductile fracture exists in the material and the structure, and when fracture occurs, the cracks propagate tortuously and do not fracture immediately. Moreover, they both have a similar structure, namely, the staggered structure. This staggered structure makes them fracture with an obvious quasi-ductile fracture behavior.

Quasi-ductile plays a multifaceted role in practical applications; for example: (1) Warning function: Quasi-ductile materials provide engineers and researchers with a certain time window to take remedial measures. Before materials approach fracture, structures can be repaired or reinforced, thereby extending the service life of the structure. (2) Guiding structural design: Understanding the behavior of materials in the quasi-ductile stage enables better prediction of crack propagation paths and rates, thus guiding structural design. (3) Optimizing material design: Researching the quasi-ductile stage allows for the identification of weaknesses and deficiencies in materials, facilitating targeted material design and improvement.

## 7. Conclusions

This study not only analyzes the fracture process of glubam but also provides a method with which to determine its fracture parameters and clarifies the characteristics of quasi-ductile fracture. The specific findings and conclusions can be summarized as follows.

(1) The fracture process of glubam can be divided into four stages, including the linear stage, softening stage, quasi-ductile stage, and failure stage. In the linear stage, the load is small and does not exceed the elastic limit of the glubam, so the load–deflection curve is linear. During the softening stage, the load–deflection curve shows non-linearity. When the fictitious crack in the T-layer reaches Δ*a_fic_*, the load reaches *P_max_*. During the quasi-ductile stage, as the T-layer is pulled apart, the load drops sharply. However, the remaining part can continue to bear the load and reach the second maximum load. Notably, this phenomenon repeats itself as the number of T-layers increases. During the failure stage, all T-layers are completely fractured, and the specimen loses its load-bearing capacity completely.
(2) In this study, the fiber tensile strength *f_t_* and fracture toughness K_IC_* of all specimens were obtained using a non-LEFM method, and a normal distribution analysis method was employed for fitting. The fracture parameters of glubam obtained during the softening stage *P_max_* are fiber tensile strength *f_t_* = *μ_f_* = 148.03 MPa and fracture toughness K_IC_* = *μ_k_* = 4.15 MPa$\sqrt{m}$. At a 96% reliability level, all the data points of the entire experiment are covered, indicating that the predicted values based on the normal distribution are reliable.
(3) The fracture parameters of glubam obtained during the quasi-ductile stage *P′_max_* are fiber tensile strength *f_t_* = *μ_f_* = 159.83 MPa and fracture toughness K_IC_* = *μ_k_* = 4.54 MPa$\sqrt{m}$. Through observation of the fracture parameters at *P_max_* and *P′_max_*, it can be seen that the fracture parameters deviate within 10% for both. Analysis of the fracture parameters, fiber distribution, and modified *a*_0_ indicates that the quasi-ductile fracture behavior of glubam results from the staggered structure formed by the L-layers and T-layers.

## Figures and Tables

**Table 1:** Mechanical properties of glubam, LBL, Douglas Fir, and WPC in parallel to the fiber direction (standard deviation).

Material	Glubam [12,14,15,16,17]	LBL [18,19,20]	Douglas Fir [13,21]	WPC [22]
Tensile strength (MPa)	80.5 (17.3)	107.7 (11.5)	49 (10.5)	11.6 (1.0)
Compressive strength (MPa)	39.5 (7.0)	56.3 (4.1)	57 (5.2)	28.1 (0.7)
Bending strength (MPa)	99 (11.9)	111.5 (8.8)	48.06 (3.3)	26.1 (1.0)
Shear strength (MPa)	15.7 (2.0)	17.5 (1.2)	11 (0.9)	8.1 (0.4)
Elastic modulus (MPa)	10,508 (1025)	11,143 (924.9)	13,000 (1872)	3000 (340)

**Table 2:** Ten groups of SEN specimens.

Group No.	*S* (mm)	*B* (mm)	*W* (mm)	*a*_0_ (mm)
G_2_-L1	112	28	28	2
G_2_-L2	112	28	28	4
G_2_-L3	112	28	28	6
G_2_-L4	112	28	28	8
G_4_-L1	192	48	48	2
G_4_-L2	192	48	48	4
G_4_-L3	192	48	48	6
G_4_-L4	192	48	48	8
G_4_-L4T1	192	48	48	10
G_4_-L8T1	192	48	48	18

## Data Availability

The data presented in this study are available on request from the corresponding author due to the potential risk of unauthorized use of original data.
